# Oral Immunogenicity of Plant-Made *Mycobacterium tuberculosis* ESAT6 and CFP10

**DOI:** 10.1155/2013/316304

**Published:** 2013-12-19

**Authors:** Elena A. Uvarova, Pavel A. Belavin, Natalya V. Permyakova, Alla A. Zagorskaya, Olesya V. Nosareva, Almagul A. Kakimzhanova, Elena V. Deineko

**Affiliations:** ^1^Institute of Cytology and Genetics, Siberian Branch, Russian Academy of Sciences, pr. Lavrentieva 10, Novosibirsk 630090, Russia; ^2^State Research Center of Virology and Biotechnology Vector, Koltsovo, Novosibirsk Oblast 630559, Russia; ^3^National Center of Biotechnology, ul. Valikhanova 13/1, Astana 010000, Kazakhstan

## Abstract

Two lines of transgenic carrot plants producing *Mycobacterium tuberculosis* proteins (ESAT6 and CFP10) have been constructed. The target proteins are present in carrot storage roots at a level not less than 0.056% of the total storage protein (TSP) for ESAT6 and 0.002% of TSP for CFP10. As has been shown, oral immunization of mice induces both the cell-mediated and humoral immunities. These data suggest that the proteins in question are appropriate as a candidate edible vaccine against tuberculosis.

## 1. Introduction

The tuberculosis caused by the bacterium *Mycobacterium tuberculosis* is currently a topical problem in many countries of the world. Recently, the number of tuberculosis cases, including fatal outcomes, has been ever increasing [1]. The recorded dynamics of tuberculosis morbidity are complicated by the spread HIV and hepatitis, ascribed by the WHO to socially significant diseases [2].

To control tuberculosis, the Bacillus Calmette-Guerin (BCG) vaccine—a live attenuated *M. bovis* strain [3]—was designed as early as the 1920s; by the end of the 20th century, about 3 billion persons worldwide had been vaccinated [4, 5]. Note that the genome of all the *M. bovis* BCG strains lacks the RD1 region, characteristic of *M. tuberculosis;* this region houses very important virulence factors, such as ESAT6 and CFP10 [6, 7]. Despite the facts that BCG as a prevention vaccine is used for protection of uninfected children and is inefficient for adults for the most abundant tuberculosis form, lung tuberculosis, BCG still remains the only available vaccine against tuberculosis [3].

For tuberculosis prevention, the efforts of numerous research groups are currently directed to design and creation of new generation antituberculosis vaccines [8]. Over ten candidate vaccines and one therapeutic vaccine against tuberculosis are now at different stages of clinical trials [2, 3]. Most of them are subunit vaccines; in general, they can be divided into two groups, namely, the vaccines for primary prevention (intended to replace BCG) and booster vaccines (used for revaccination). The booster vaccines are necessary to prevent reactivation of the pathogen during latent tuberculosis [9].

Secreted *M. tuberculosis* proteins are used in designing booster vaccines; the most important of them are the proteins that interfere with the integral components in the protection against tuberculosis, that is, those able to induce a strong T-cell response and *γ*-interferon secretion [10–12]. The ESAT6 and CFP10 proteins are among the key cell virulence factors of *M. tuberculosis.* They do induce a strong T-cell response and, presumably, are involved in the lysis of the host cell membrane or the overall host cell. These proteins are secreted by the ESX-1 system, comprising at least ten genes (*Rv3868*–*Rv3877*). Under in vitro conditions, the purified recombinant ESAT6 and CFP10 form heterodimeric complexes [13]. It has been shown that the recombinant ESAT6 and CFP10 are able to form a homodimeric complex in yeast [14].

Since the tuberculosis pathogen is an airborne bacterium, the organism's immune response, both mucosal and systemic, can be induced via mucosa of the warm-blooded hosts. Thus, design of the vaccine intended for induction of the immune response at the level of mucosae is a doubtlessly promising challenge towards candidate vaccines against tuberculosis. One of the promising approaches here is associated with the transgenic plants producing protective antigens, the so-called edible vaccines [15].

The studies on antituberculosis vaccines involving plant expression systems and transferring the sequence of *M. tuberculosis* gene *esat6* to plants are rather few [16–21]. Fusion of the ESAT6 antigen with other tuberculosis antigens, such as Ag85B or Mtb72F [17, 18, 20, 22], and use of various adjuvants, such as CTB, LTB, LipY, or ELP [17, 19, 21] with further expression in various plant species (*Arabidopsis thaliana,* tobacco, and lettuce), have been attempted. For this purpose, various plant expression systems have been utilized, namely, agroinfiltration [16, 18] as well as nuclear [17, 19, 20, 22] and chloroplast [21] transformations.

The goal of this work was to construct the transgenic carrot plants producing the *M. tuberculosis* ESAT6 or CFP10 proteins and to analyze their immunogenicity in laboratory animals (mice) using an oral antigen delivery.

## 2. Materials and Methods

### 2.1. Isolation of *M. tuberculosis* Genomic DNA

The genomic DNA was isolated from the biomass of an *M. tuberculosis* clinical isolate recovered from a tuberculosis patient using a DNeasy Blood and Tissue Kit (QIAGEN, Germany) according to the manufacturer's protocol.

### 2.2. Amplification of *esat6* and *cfp10* Gene Sequences and Their Cloning into the Binary Vector pBI121

The following primers were used for amplifying the genes *esat6* and* cfp10* using the *M. tuberculosis* genomic DNA as a template: esat-upper 5′-GCTCTAGAATGACAGAGCAGCAGTGGAATTTCGCGG-3′ and esat-low 5′-CGGGATCCCTATGCGAACATCCC-3′ for the *esat6* gene and for the *cfp10* gene, cfp-upper 5′-GCTCTAGAATGGCAGAGATGAAGACC-3′, and cfp-low 5′-CGGGATCCGAATTCTCAGAAGCC-3′.

**Figure 1:**
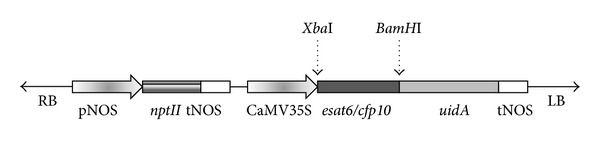
Scheme of the T-region in binary plasmid pBI121, carrying the sequences of *M. tuberculosis* target genes *esat6* or *cfp10.* Designations: RB and LB, border repeats flanking the T-region; pNOS: promoter of the nopaline synthase gene of *A. tumefaciens* Ti plasmid; *nptII: E. coli* neomycin transferase II gene; tNOS: terminator of the nopaline synthase gene of *A*.* tumefaciens* Ti plasmid; CaMV35S: promoter of the cauliflower mosaic virus 35S RNA gene; *esat6/cfp10:* sequence of the target *M. tuberculosis esat6 *or* cfp10 *gene; *uidA:* sequence of the gene encoding the enzyme *β*-glucuronidase. Arrows denote *Bam*HI and *Xba*I restriction sites.

After amplification, the fragments of *esat6 *(288 bp) and* cfp10 *(303 bp) genes were hydrolyzed with the restriction endonucleases *Xba*I and *BamH*I, purified using a GenElute HP Plasmid Midiprep kit (Sigma-Aldrich, United States), and ligated with the pBI121 preliminary hydrolyzed at the same sites. The resulting reaction mixture was used to transform *E. coli* strain DH10B.  Figure 1 shows the map of the plasmid carrying the target genes (*esat6* or *cfp10*).

### 2.3. Construction of Transgenic Carrot Plants

The carrot callus tissues induced from carrot (*Daucus carota *L. cultivar Nantskaya) zygotic embryos were transformed by agrobacterial transfer [23]. The transformants were selected using the selective media supplemented with the antibiotic kanamycin (100 g/L). The kanamycin-resistant plants were grown in a hydroponic greenhouse to development of storage roots.

### 2.4. Plant DNA Isolation

The genomic DNA was isolated from carrot plants using an AxyGen (United States) DNA isolation kit according to the manufacturer's protocol.

The presence of target genes (*esat6* or *cfp10*) in the genomes of the constructed transgenic carrot plants was verified by PCR with the corresponding primers mentioned above using the DNA isolated from the leaves of transformed carrot plants (1 *μ*g) as a template.

PCR was conducted in 20 *μ*L of the buffer containing 67 mM Tris-HCl (pH 8.9), 16 mM (NH_4_)_2_SO_4_, 1.5 mM MgCl_2_, 0.01% Tween 20, 200 *μ*M of each dNTP, 1 AU of *Taq* polymerase (SibEnzim, Novosibirsk, Russia), and 100 *μ*M of each primer. PCR was performed after preliminary heating at 94°C for 2 min according to the following scheme: 34 cycles of 15 s at 94°C, 30 s at 55°C, and 45 s at 72°C and final synthesis for 2 min at 72°C. The PCR products were analyzed by electrophoretic separation in 2% agarose gel.

### 2.5. Production and Purification of Recombinant rESAT6 and rCFP10

The recombinant rESAT6 and rCFP10 (fused with GST peptide), used in the experiments as a control, were produced beforehand in *E. coli* strain BL21 (DE3). The recombinant proteins were affinity purified on Glutathione Sepharose 4B (Pharmacia, Sweden) as earlier described [24].

### 2.6. Producing Carrot Storage Root Extracts

Specimens of carrot storage roots (6 g) sampled from at least six plants were minced, ground in a mortar in liquid nitrogen, and homogenized in 20 mL of the buffer containing 0.1 M Tris-HCl (pH 7.5), 12 mM 2-mercaptoethanol, 1% SDS, 10 mM EDTA, and 3 mM PMSF in the presence of insoluble PVP (2.5% relative to the volume of extraction buffer). The homogenate was centrifuged for 20 min at 10 000 rpm. The supernatant was supplemented with PVP (2.5–5.0% relative to the volume of extraction buffer) and centrifuged for 20 min at 10 000 rpm. The proteins were precipitated with 10% solution of trichloroacetic acid. The concentrations of purified rESAT6 and rCFP10 recombinant proteins as well as the TSP in carrot storage roots were determined according to Bradford [25]. Optical density of solutions was recorded in an Eppendorf BioPhotometer plus (Eppendorf, Germany).

### 2.7. Producing Mouse Polyclonal Antibodies to the Recombinant Antigens rESAT6 and rCFP10

Female ICR mice (ten animals) at an age of 1.5 months were intraperitoneally immunized with solutions of the recombinant proteins rESAT6 or rCFP10 according to the following scheme: day 1, 50 *μ*g of antigen (rESAT6 or rCFP10) in 100 *μ*L CFA; day 14, 25 *μ*g antigen in 100 *μ*L FIA; and day 28, 25 *μ*g of antigen in 100 *μ*L PBS (1.7 mM KH_2_PO_4_, 5.2 mM Na_2_HPO_4_, and 150 mM NaCl; H_2_O mQ, pH 7.4). On day 35, the animals were euthanized and bled to isolate the serum. The sera were obtained by natural clotting with subsequent elimination of the blood elements by centrifugation at 3000 rpm for 10 min. The serum specimens were cryopreserved at −80°C to further assay for the level of antibodies to the recombinant antigens rESAT6 or rCFP10 by solid-phase enzyme immunoassay [26].

### 2.8. Producing Rabbit Polyclonal Antibodies to the Recombinant Antigens rESAT6 and rCFP10

The *Seryi velikan* strain rabbits at an age of 6 months were immunized according to the following scheme: day 1, 100 *μ*g of antigen (rESAT6 or rCFP10) with 500 *μ*L of CFA intracutaneously; day 14, 100 *μ*g of antigen with 500 *μ*L of FIA subcutaneously; and days 28 and 42, 100 *μ*g of antigen subcutaneously. Animals were bled (5–10 mL) from the auricular vein on days 28, 42, and 56. The sera were obtained by natural clotting with subsequent elimination of the blood elements by centrifugation at 3000 rpm for 10 min. The specimens were frozen and stored at −20°C until being assayed.

### 2.9. Detecting Recombinant Proteins in the Root Tissues of Transgenic Carrot Plants

The antigens rESAT6 and rCFP10 were detected by dot blot and Western blot assays, respectively, according to the standard protocols [27]. Chemiluminescence reaction was conducted using Pierce ECL Western Blotting Substrate (Pierce, United States).

### 2.10. Assaying Immunogenic Characteristics of the Transgenic Carrot Carrying the Recombinant Antigens rESAT6 or rCFP10

**Figure 2:**
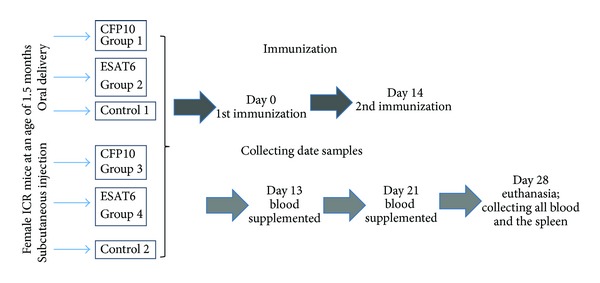
Scheme of the immunization protocol. *Estimating the cell-mediated component of immunity.* The cell-mediated component of immunity was assessed by LBTT. The peripheral blood lymphocytes were isolated from the collected blood with anticoagulant and splenocytes, from the homogenated spleen cell suspension. The blood with anticoagulant as well as spleen cell suspension was layered onto Histopaque-1119 gradient (Sigma-Aldrich, United States) with subsequent centrifugation (15 min at 500 rpm). The mononuclear and splenocyte suspensions were washed with and suspended in RPMI 1640 (2 mM *L*-glutamine, 80 mg/L gentamicin, and 10% fetal serum). A concentration of 5 × 10^5^ cells/mL was used for LBTT.

Scheme of the immunization protocol is depicted in Figure 2. Female ICR mice at an age of 1.5 months were used in the experiment. The animals were divided into six groups: group 1, oral delivery of the ESAT6 antigen; group 2, oral delivery of the CFP10 antigen; group 3, subcutaneous injection of rESAT6 solution; group 4, subcutaneous injection of rCFP10 solution; group 5, control 1 (nontransgenic carrot, orally); and group 6, control 2 (PBS solution, subcutaneously). Double oral immunization of animals was performed after an adaptation period with a 2-week interval (5 g per animal at days 0 and 14 as a single dose) after 12 h fast or subcutaneously with solutions of the recombinant antigens rESAT6 or rCFP10 (100 ng of antigen in a volume of 50 *μ*L). After 6–10 h, the amount of eaten carrot was estimated. Animals of all groups consumed the carrot completely. Then the animals were fed standard diet.

On days 13 and 21 of experiment, the animals of all six groups were bled from the retroorbital sinus. The blood supplemented with anticoagulant (5% sodium EDTA solution) was used to isolate peripheral blood leukocytes. The blood without anticoagulant was used to obtain the blood sera by natural clotting with subsequent purification from the blood cell elements by centrifugation at 3000 rpm for 10 min. The serum specimens were cryopreserved in Eppendorf tubes at −80°C.

On day 28 of observation, the animals were euthanized by cervical dislocation to collect all blood and obtain the serum and leukocytes. The spleen was excised under aseptic conditions and placed into the RPMI 1640 nutrient medium (2 mM *L*-glutamine, 80 mg/L gentamicin, and 5% fetal bovine serum) for further isolation of splenocytes and lymphocyte blast-transformation test (LBTT).

For nonspecific cell stimulation, 0.5 *μ*g/mL phytohemagglutinin (PHA; Sigma, United States) was used at a dose of 20 *μ*L per one plate well (positive control); for specific stimulation, 20 *μ*L/well of the corresponding antigen (rESAT6 or rCFP10) at a concentration of 10.0 *μ*g/mL was added to each well and the control wells were supplemented with 20 *μ*L of culture medium (negative control). Proliferative activity was assessed with the help of the MTT dye (TASC MTT Assays, R&D Systems, United States) according to the manufacturer's protocol. Optical density (OD) was recorded at a wavelength of 630 nm in a Bio-Tek FL600 vertical automated fluorescence reader (Bio-Tek Instruments, United States). Cell proliferative activity was assessed according to stimulation index. The index was calculated as the ratio of the mean absolute OD values for the stimulated cells to the mean absolute OD values for the unstimulated cells.

### 2.11. Estimating the Humoral Immunity Component by EIA

The antibodies were detected in the blood specimens by solid-phase enzyme immunoassay [26].

### 2.12. Statistical Processing

Statistical analysis was performed by calculating the average values and the average deviation (M + m) using Microsoft Excel.

## 3. Results

### 3.1. Assessing the Transgenic Status of the Produced Carrot Transformants

To validate a transgenic nature of the constructed carrot transformants, each storage root was assayed by PCR for the presence of the DNA fragments with the lengths corresponding to the expected sizes of target *esat6* and *cfp10* fragments in the carrot genomic DNA.

**Figure 3:**
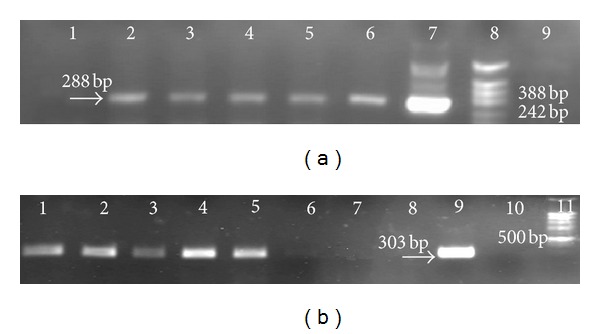
Electrophoretic pattern (1.5% agarose gel) of the PCR products amplified using the primers specific for the *esat6 *(a) gene: (1) DNA of nontransgenic carrot plant; (2–6) DNAs of the carrot plants transformed with the plasmid pBI121 carrying the *esat6 *gene; (7) DNA of the plasmid pBI121 carrying the *esat6 *gene; (8) DNA marker (SibEnzim, Novosibirsk, Russia), pBluescript II SK(+) hydrolyzed with the *Msp*I restriction endonuclease; and (9) negative control (without DNA template); for* cfp10(b)*: (1–7) DNAs of the carrot plants transformed with the plasmid. pBI121 carrying the *cfp10 *gene; (8) DNA of nontransgenic carrot plant; (9) DNA of the plasmid. pBI121 carrying the *cfp10 *gene; (10) negative control (without DNA template); and (11) DNA marker (SibEnzim, Novosibirsk, Russia).

The presence of the amplified fragments in lanes 2–6 (Figure 3) matching the length of the expected PCR fragment of the plasmid (288 bp) suggests that the target *esat6* gene has integrated into the nuclear genome of the studied plants. The presence of the amplified fragments in lanes 1–5 (Figure 3) matching the length of the expected PCR fragment of the plasmid (303 bp) suggests that the target *cfp10* gene has integrated into the nuclear genome of the studied plants.

### 3.2. Enzyme Immunoassay of the Extracts of Transgenic Carrot Storage Roots

**Figure 4:**
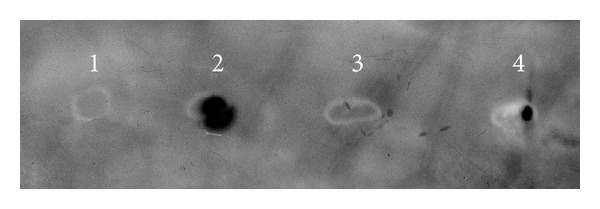
Enzyme immunoassay (dot blot hybridization) for the CFP10 content in carrot storage roots: (1) PBS buffer (negative control 1), (2) rCFP10, 360 pg (positive control), (3) extract of nontransgenic carrot storage roots (negative control 2), and (4) extract of the transgenic carrot storage roots (1 mkl) carrying the *cfp10* gene.

Figure 4 shows results of the assay for CFP10 antigen in the extracts of transgenic carrot storage roots by dot blot hybridization. The presence of signals at dot 4 (Figure 4) suggests that the tissues of transgenic carrot storage roots contain a functional CFP10 protein capable of binding with the corresponding polyclonal antibodies to the rCFP10, produced by *E. coli*.

**Figure 5:**
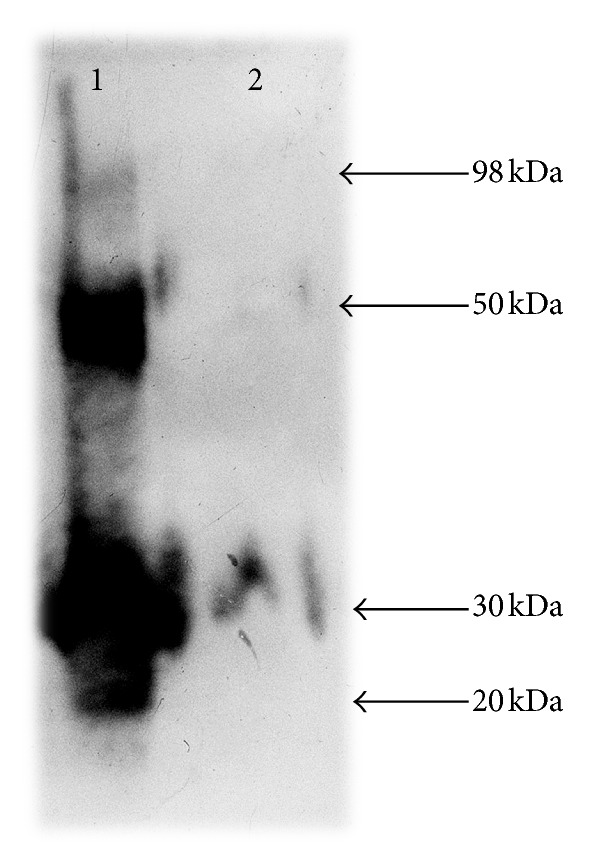
Enzyme immunoassay (Western blot hybridization) for the ESAT6 content in carrot storage roots: (1) extract of the transgenic carrot storage roots (15 mkl) carrying the *esat6* gene and (2) recombinant ESAT6 protein (100 ng); molecular weights of the visualized fragments are shown to the right.

Figure 5 shows the EIA data for the recombinant ESAT6 antigens synthesized in transgenic carrot tissues (lane 1) and *E. coli* (lane 2). Four fragments of different lengths (98, 50, 30, and 20 kDa) that bind with the serum antibodies to the rESAT6 produced in *E. coli* are detectable in lane 1.

According to the EIA data, the accumulation level of the studied recombinant proteins in the storage roots of transgenic carrot plants is 0.056% TSP for rESAT6 (TSP is 1190 ng/mkl) and 0.024% TSP for rCFP10 (TSP is 1500 ng/mkl).

### 3.3. Assessing the Cell-Mediated Immunity in Laboratory Animals after Oral Immunization

**Figure 6:**
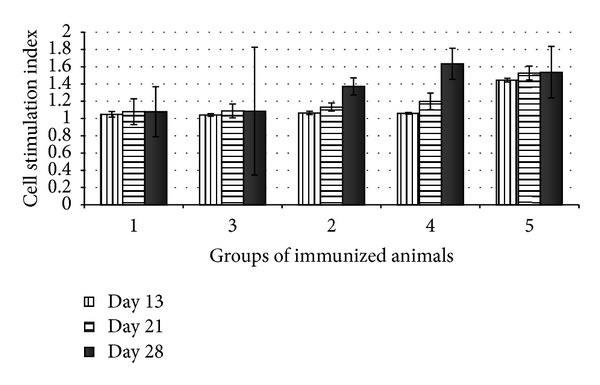
LBTT for the immunized animals after stimulation with the mitogen PHA: (1) animals orally immunized with the recombinant carrot (5 g) producing the ESAT6 protein; (2) animals orally immunized with the recombinant carrot (5 g) accumulating producing the CFP10 protein; (3) animals immunized with the rESAT6 protein (subcutaneously 100 ng); (4) animals immunized with the rCFP10 protein (subcutaneously 100 ng), and (5) animals of the pooled control group (control 1—nontransgenic carrot, orally, and control 2—PBS solution, subcutaneously).

Data obtained using LBTT test were prepared by combining statistics of spleen and blood lymphocytes.  Figure 6 shows the data on nonspecific stimulation of lymphocytes with the mitogen PHA. It is evident that the cell stimulation index almost does not grow on day 28 in the animals of groups 1 and 3 (immunized with ESAT6), which suggests a certain suppression of the lymphocytes of immunized animals as compared with the control group. These results indicate likely toxicity of ESAT6 for the cell-mediated immunity.

**Figure 7:**
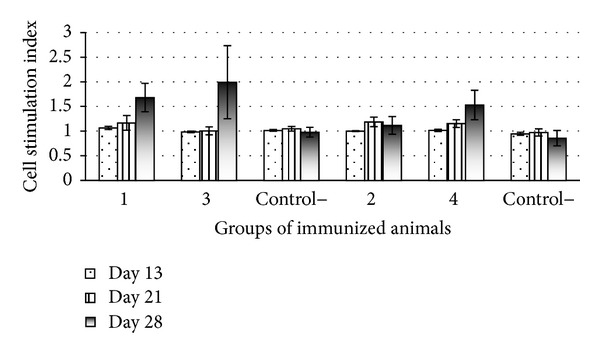
LBTT for immunized animals after stimulation with the recombinant antigens rESAT6 or rCFP10: (1) animals orally immunized with the recombinant carrot (5 g) producing the ESAT6 protein; (2) animals orally immunized with the recombinant carrot (5 g) producing the CFP10 protein; (3) animals immunized with the rESAT6 protein (subcutaneously 100 ng); (4) animals immunized with the rCFP10 protein (subcutaneously 100 ng), and (control–) negative control (control 1—nontransgenic carrot, orally, and control 2—PBS solution, subcutaneously).

Assessment of the specific immune response of the cells of immunized animals to in vitro stimulation with the recombinant antigens ESAT6 or CFP10 has demonstrated a statistically significant increase in the lymphocyte activity on day 28 after immunization (Figure 7). The lymphocyte stimulation was most pronounced in the groups immunized with ESAT6 (groups 1 and 3); note that the cell stimulation index in group 3, immunized with the recombinant protein rESAT6, was higher as compared with that in group 1, immunized with the transgenic carrot producing this protein.

### 3.4. Assessing the Humoral Immunity in Laboratory Animals after Oral Immunization

**Figure 8:**
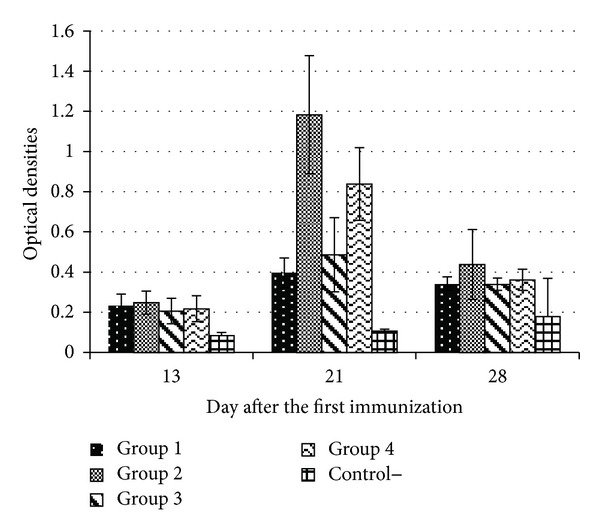
Antibody level in the blood sera of immunized animals: (1) group of the mice immunized with the carrot (5 g) producing ESAT6 protein; (2) group of the mice immunized with the carrot (5 g) producing CFP10 protein; (3) group of the mice immunized with rESAT6 (subcutaneously 100 ng); (4) group of the mice immunized with rCFP10 (subcutaneously 100 ng) (control 1—nontransgenic carrot, orally, and control 2—PBS solution, subcutaneously).

Assay for the level of antibodies (Figure 8) in the blood serum of the immunized mice has demonstrated induction of the IgG specific for the recombinant antigens rESAT6 or rCFP10 in all four groups of immunized animals.

Table 1 lists the data on the dynamics of animals displaying a positive EIA response (i.e., the number of animals with the OD values in the blood serum exceeding the calculated threshold OD). On days 21 and 28, almost all animals displayed a positive response as estimated according to antibody production. It should be emphasized that the level of IgG accumulation in the blood of immunized animals reached its maximum on day 21 and decreased by day 28. However, the IgG level recorded in the blood of experimental animals on day 28 was higher than the corresponding initial level, recorded on day 13 (Figure 8).

## 4. Discussion

There is a need for alternative routes of vaccine delivery targeting the mucosa which is the site of invasion of *Mycobacterium tuberculosis*. In order to combat the TB disease with a safer vaccine, subunit vaccines are being pursued with recombinant TB antigens. Oral delivery of antigens bioencapsulated in plant cells eliminates the highly expensive purification, cold storage, transportation, and sterile injections, significantly reducing their cost. An attractive feature of the transgenic plants is the fact that even when target antigene is expressed at a low level, specific immune response can be formed.

Several research teams have demonstrated feasibility of production of *M. tuberculosis* antigens in plant expression systems. The ESAT6 antigen fused with *E. coli* heat-labile toxin B subunit has been shown to accumulate in tissues of transgenic *A. thaliana* [19]. It has been demonstrated that both components of the hybrid protein retain their antigenic structures and are able to form pentamers. Analysis of the immune responses to an oral delivery of these antigens in mice has made it evident that the delivered antigens are able to induce Th1 and Th2 immune responses [19, 28].

The genes encoding *M. tuberculosis* ESAT6 and Ag85B antigens, both separately and in a fused form (ESAT6-Ag85B), have been transferred to *Lactuca sativa *L. plants [20] and hairy root culture of *Cichorium intybus *L. var. *foliosum* Hegi [22]. However, the authors have not assayed accumulation level of the target proteins in plant tissues and their immunogenicity.

The fusion protein comprising the *M. tuberculosis* antigens Ag85B and ESAT6 fused with elastin-like peptide has been produced in transgenic tobacco plant tissues [17]. Immunogenicity (T cell-mediated immune response) of the studied recombinant fusion protein has been confirmed by the authors in tests with mice and pigs. As has been shown, the B- and T-cell epitopes of Ag85B and ESAT6 antigens within complex fusion proteins produced in plant expression systems are not subject to modifications and retain their ability to bind the corresponding antigens [17].

In a transient expression system, the accumulation level of recombinant ESAT6 antigen amounted to 0.5–1.0% TSP in fresh *Nicotiana tabacum* leave tissue [16]. An overexpression (not less than 800 mg/kg fresh tobacco leaves) of Ag85B and ESAT6 antigens synthesized in plant tissue both separately and as the fusion protein was recorded by Dorokhov et al. [18]. In order to elevate the accumulation of recombinant protein in plant tissues, tuberculosis antigens were for the first time expressed in chloroplasts [21]. The level of ESAT6 and the fusion protein Mtb72F (Mtb32–Mtb39) fused to CTB and LipY reached 0.75% TSP in the leaves of transplastome lettuce plants and 1.2–7.5% TSP in tobacco plants.

A large part of these works has been performed in model plant species, inappropriate for human consumption and, correspondingly, not promising for further application as edible vaccines. Despite that the immunogenicity of produced recombinant proteins has been tested in the experiments with laboratory animals only in two works [17, 28], these data make it evident that *M. tuberculosis* antigens can be successfully expressed in plant cells retaining their antigenic structure and can accumulate in plant tissues at the level sufficient for establishment of the immune response.

In this work, we have pioneered in demonstrating that expression of the *M. tuberculosis* ESAT6 and CFP10 antigens in carrot storage roots as well as verifying their immunogenicity after an oral delivery in animal (mouse) model. We have constructed two types of transgenic carrot plants, namely, carrying the *M. tuberculosis* genes *esat6* and *cfp10.* EIA has shown accumulation of the target proteins in the storage roots of transgenic carrot plants. Induction of the cell-mediated and humoral immunities by oral administration of the recombinant antigens in question to experimental animal has been shown. Induction of the immune response by oral administration of the recombinant CFP10 antigen synthesized in the storage root tissues of transgenic carrot plants has been demonstrated for the first time.

It is known that proteins can be subject to posttranslational modification (glycosylation, hydrolysis, cleavage, or polymerization) in a plant expression system [29]. When visualizing the ESAT6 protein by Western blot hybridization, several bands were detected in the lane containing the total protein extract of transgenic carrot storage root (Figure 5). The patterns of the corresponding lanes suggest that the ESAT6 protein expressed in the carrot storage roots is likely to form homodimeric complexes and is present in several forms. Since the polyclonal serum for binding the studied protein (in Western blot assay) was obtained using the recombinant protein rESAT6, it is logical to assume that all the molecules containing ESAT6 polypeptide will be visualized when analyzing the total protein extract. The data on the composition and molecular weights of the peptides contained in the fusion protein make it possible to predict the molecular weights of the visualized peptides after their cleavage or polymerization. The fragment with a molecular weight of about 98 kDa corresponds to the weight of the fused polypeptide ESAT6-*β*-glucuronidase. The remaining fragments (50, 30, and 20 kDa) are likely to be ESAT6 homopolymers formed after release of *β*-glucuronidase, which agrees well with the known data [13, 14]. This suggests that the ESAT6 expressed in carrot cells retains its antigenic characteristics. However, the expression levels of ESAT6 and CFP10 antigens in the storage roots of constructed transgenic carrot plants are still insufficient to design a commercial product. To improve the transcriptional activity of the transgenic constructs, it is further suggested to optimize nucleotide context of the promoter regions [30].

Analysis of the immunogenic properties of transgenic carrot storage roots suggests that the recombinant ESAT6 or CFP10 antigens expressed in plants are able to induce both the cell-mediated and humoral immune responses being orally delivered to a warm-blooded organism. It has been demonstrated that ESAT6 is toxic for the tested animals (Figure 6); in addition, we have found (unpublished data) that this protein may have a negative effect at the stage of regeneration of transformed plants under in vitro conditions (causing various morphological abnormalities in regenerants). CFP10 is chaperone to ESAT6, so these two proteins function in cooperation. Further promising direction in the work with these antigens can be in developing the recombinant fusion protein, that in our opinion can reduce the toxic effects of ESAT6-antigen. Thus, our further task is to decrease the toxic effect, elevate the expression level, and increase immunogenicity of the candidate edible vaccine against tuberculosis.

## 5. Conclusions

This paper for the first time describes the data demonstrating that transgenic carrot, whose storage roots may be consumed without any heat processing, may form the platform for producing the immunogenic mycobacterial proteins ESAT6 and CFP10 and designing an edible antituberculosis vaccine.

## Figures and Tables

**Table 1:** Dynamics of positive response assessed by EIA.

	Day 13	Day 21	Day 28
Group 1	2	5	6
Group 3	1	6	6
Group 2	3	6	6
Group 4	2	6	6
Control	0	0	0

## Abbreviations

CFA: Complete Freund's adjuvant
CTB: Cholera toxin B subunit
EDTA: Ethylenedinitrilo-tetraacetic acid
ELP: Elastin-like peptide
FIA: Freund's incomplete adjuvant
GST: Glutathione S-transferase
LTB: B subunit of *Escherichia coli* heat-labile enterotoxin
LipY: A cell wall protein
OD: Optical density
PMSF: Phenylmethanesulfonyl fluoride
PVP: Polyvinylpyrrolidone
SDS: Sodium dodecyl sulfate
TSP: Total soluble protein.
